# Contribution of promoter DNA sequence to heterochromatin formation velocity and memory of gene repression in mouse embryo fibroblasts

**DOI:** 10.1371/journal.pone.0217699

**Published:** 2019-07-03

**Authors:** Patricia A. Vignaux, Celyn Bregio, Nathaniel A. Hathaway

**Affiliations:** 1 Division of Chemical Biology and Medicinal Chemistry, Center for Integrative Chemical Biology and Drug Discovery, UNC Eshelman School of Pharmacy, Chapel Hill, North Carolina, United States of America; 2 Curriculum for Genetics and Molecular Biology, University of North Carolina, Chapel Hill, Chapel Hill, North Carolina, United States of America; Texas A&M University, UNITED STATES

## Abstract

Durable gene silencing through the formation of compact heterochromatin domains plays a critical role during mammalian development in establishing defined tissues capable of retaining cellular identity. Hallmarks of heterochromatin gene repression are the binding of heterochromatin protein 1 (HP1), trimethylation of lysine 9 on histone H3 (H3K9me3) and the methylation of cytosine residues of DNA. HP1 binds directly to the H3K9me3 histone modification, and while DNA methyltransferases have been found in complex with histone methyltransferases and HP1, there remains much to be known about the relationship between DNA sequence and HP1 in differentiated mammalian cells. To further explore this interplay in a controlled system, we designed a system to test the effect of promoter CpG content on the formation kinetics and memory of an HP1-mediated heterochromatin domain in mouse embryo fibroblasts (MEF)s. To do this, we have constructed a side-by-side comparison of wild-type (CpGFull) and CpG-depleted (CpGDep) promoter-driven reporter constructs in the context of the Chromatin *in vivo* Assay (CiA), which uses chemically-induced proximity (CIP) to tether the chromoshadow domain of HP1α (csHP1α) to a fluorescent reporter gene in a reversible, chemically-dependent manner. By comparing the response of CpGFull and CpGDep reporter constructs, we discovered that the heterochromatin formation by recruitment of csHP1α is unaffected by the underlying CpG dinucleotide content of the promoter, as measured by the velocity of gene silencing or enrichment of H3K9me3 at the silenced gene. However, recovery from long-term silencing is measurably faster in the CpG-depleted reporter lines. These data provide evidence that the stability of the HP1 heterochromatin domain is reliant on the underlying DNA sequence. Moreover, these cell lines represent a new modular system with which to study the effect of the underlying DNA sequences on the efficacy of epigenetic modifiers.

## Introduction

The mammalian genomic landscape can be broadly divided into two regions defined by chromatin accessibility and a number of epigenetic marks. Active genes reside in a more open and accessible euchromatin compartment, allowing for facile transcription factor binding and transcription of RNA, while inactive genes are typically found in condensed heterochromatin. Gene expression programs, which are carefully maintained through selective gene repression by heterochromatin pathways, are carefully timed during development and then faithfully preserved in defined tissues. Multiple distinct epigenetic pathways are in place to ensure that heterochromatinized regions of the genome remain transcriptionally silenced after successive rounds of cell division. The heterochromatin protein-1 (HP1) pathway relies on a feedforward method of propagation, where the chromodomain of HP1 binds a histone H3 tri- or di-methylated lysine (H3K9me2/3), then recruits histone methyltransferases (HMTs) like SET domain bifurcated 1 (SETDB1) or suppressor of variegation 3–9 homolog 1 or 2 (SUV39H1/2) to methylate the same residue on neighboring nucleosomes [1–6]. HP1 and H3K9me2/3 are normally found at the pericentromere and also along the chromosomes at developmentally-regulated genes in somatic cells [7,8]. Heterochromatin is also observed transiently at the sites of double-stranded breaks and is important for the Ataxia Telangiectasia Mutated (ATM) double-stranded break repair pathway [9,10]. These markers of heterochromatin, like many other epigenetic modifications, have been found perturbed in human disease [11–14]. Heterochromatin patterning is also very closely connected to another well-studied mark of heterochromatin that is intrinsically tied to the underlying DNA sequence, DNA methylation.

DNA methylation was one of the first epigenetic control mechanisms discovered to silence gene expression. Even before the characterization of gene promoters, it was shown that methylated DNA adjacent to a gene of interest would repress genes [15]. Cytosine residues in the context of a CpG dinucleotide are modified by the addition of a methyl group to the 5’ carbon of the cytosine nucleotide base. This mark is faithfully propagated from parent to daughter strand during DNA replication by a maintenance DNA methyltransferase (DNMT1), though it has been shown that the *de novo* DNA methyltransferases DNMT3a and DNMT3b are indispensable for DNA methylation in mouse embryonic stem cells (mESCs) and DNMT3b is indispensable in mouse embryo fibroblasts (MEFs) [16–18]. In the early 1980s, it was discovered that there were regions of the genome that were enriched for the CpG dinucleotide but were, unlike the rest of the mammalian genome, not methylated [19]. A subset of these CpG islands were methylated during development, while another set were found to be in promoters of house-keeping genes and are kept free from methylation in differentiated cells [20,21]. Appropriate DNA methylation is part of normal cellular differentiation during development. DNA methylation is required to silence repetitive genomic elements, for X-inactivation in females, for appropriate imprinting of parental alleles, and to maintain proper compaction and genomic integrity during mitosis [16,22,23]. However, DNA can become inappropriately methylated in diseases, such as cancer, and disrupt gene expression programs of the cell [24–30].

While it is well documented that these two gene-silencing pathways work in tandem, often overlapping in silenced regions of the mammalian genome, there is some evidence that the two might also have interdependent roles, as is found in other organisms [8,31–36]. For example, during DNA replication DNMT1 works in conjunction with a RING finger type E3 ubiquitin ligase UHRF1 that can bind both hemi-methylated DNA and H3K9me3 to maintain DNA methylation in dividing cells [32,37]. However, it has been complicated to examine causal relationships due to the lack of tools that can study kinetics in the presence or absence of CpG methylation sites. In order to determine if an HP1-mediated heterochromatin domain is affected by the underlying DNA sequence, or if it can persist in the absence of DNA methylation over repeated cell divisions, we devised a system to explore the behavior of an HP1-mediated domain in the presence or absence of CpG dinucleotides in the immediate promoter regions of a knock-in reporter. This system uses chemically-induced proximity (CIP) to reversibly tether the chromoshadow domain of HP1α (csHP1α) upstream of the transcription start site of a reporter gene. The chromoshadow domain of HP1 is responsible for binding other proteins, including other HP1 monomers and HMTs, and can induce a heterochromatin domain as sufficiently as full-length HP1α [38–40]. By tethering an ectopic csHP1α to the gene locus, we were able to chemically initiate a heterochromatin domain across the promoter and reporter gene body. In this study, we created reporter cassettes with one of two promoters driving a nucEGFP reporter: a wild-type CMV-EF1α promoter (CpGFull) or a “CpG depleted” version of the promoter (CpGDep) that is devoid of all CpG dinucleotides [41].

In order to explore the silencing dynamics and the heterochromatin memory attributed to CpG dinucleotides, we captured gene expression from these transgenes by measuring GFP levels. We were surprised that despite the differences in the underlying DNA sequences, initial experiments showed both the CpGFull and CpGDep promoters were silenced at roughly the same velocity, as defined by hours of CIP-rapamycin csHP1α recruitment required before measurable GFP repression and H3K9me3 enrichment. Upon release of short-term csHP1α recruitment, the CpGDep and CpGFull cassettes both rapidly lost the heterochromatin domain and re-expressed GFP within six days. However, after an extended period of csHP1α-induced heterochromatin, the CpGFull promoter alone was able to maintain a silenced state after csHP1α release by CIP washout.

## Materials and methods

### Promoters

The CpGDep promoter was obtained from Dr. Michael Rheli, in the plasmid pCpGL 865. The sequence was cloned into an expression plasmid containing a nucEGFP reporter gene with homology to a region of the β-globin locus kilobases from any other gene and relatively devoid of known epigenetic marks; a locus selection that was inspired by others’ previous work [42]. The CpGFull promoter was stitched together by PCR and was designed to mimic the CpGDep promoter as much as possible in length and nucleotide composition. All primers used in this project are reported in Table 1. Homology arm sequences are reported in S1 Table.

**Table 1:** 

Primer Name	Primer Sequence	Primer Use
PVP136	GATGTGCGCTCTGCCCACTGAACTCCCATTGACGTCAATGGGG	Inside reverse sewing primer to create CpGFull promoter for infusion into N261
PVP137	CCCCATTGACGTCAATGGGAGTTCAGTGGGCAGAGCGCACATC	Inside forward sewing primer to create CpGFull promoter for infusion into N261
PVP138	TCGAGGGATCAAGCTTCGAAAAAGAACGTTCACGGCGA	Outside reverse sewing primer to create CpGFull promoter for infusion into N261
PVP164	GGGGCCGGCCGGATCCGAGTCAATGGGAAAAACCCATTGG	Amplify CpGFree promoter for infusion into N261
PVP165	ATTACTCGAGGGATCTTAATTAAGAATGTTCACAGAGACTACTGCAC	Amplify CpGFree promoter for infusion into N261
PVP168	GGGGCCGGCCGGATCCTAGTTATTAATAGTAATCAATTACGGGGT	Amplify CpGFull promoter for infusion into N261
PVP169	ATTACTCGAGGGATCTTAATTAACGAAAAAGAACGTTCACGGCGA	Amplify CpGFull promoter for infusion into N261
sgRNA PV001	CACCGTGTCTGCTCTGAACTGAAA	Oligo to create sgRNA and anneal into CRISPR delivery plasmid
sgRNA PV002	AAACTTTCAGTTCAGAGCAGACAC	Oligo to create sgRNA and anneal into CRISPR delivery plasmid
PVP183	GACCACATGAAGCAGCACGACTTC	Primer inside of nucEGFP to determine successful CRISPR insertion
PVP083	GCGGACTAGTCCCGGGGCGATCGCACTGACCTCTGGGGCTATACTG	Forward primer to amplify Balb/C homology arm 2 for infusion into N272
PVP084	ATTCCTGCAGCCCGGTTGGCTACTCCTTAAGGTATAAATTGAAG	Reverse primer to amplify Balb/C homology arm 2 for infusion into N272
PVP085	GGGGCCGGCCGGATCGCGGCCGCGACCATGGTGTCCATGTCATACAG	Forward primer to amplify Balb/C homology arm 2 for infusion into ____
PVP086	ATTACTCGAGGGATCCATGCATACTGAAAAGGGAGGGATTTCTAGC	Reverse primer to amplify Balb/C homology arm 2 for infusion into ____
PVP185	TGCACATCAGTATGGCTTTTGAGGC	Primer upstream of 5’ homology sequence to determine successful CRISPR insertion
PVP228	TAATTAYGGGGTTATTAGTTTATAGTTTATATATGGAG	Bisulfite sequencing primer for CpGFull promoter
PVP234	TATATACRATTCTCCCCCACCCTC	Bisulfite sequencing primer for CpGFull promoter
489_2s	GCGCACCATCTTCTTCAAGG	ChIP primer in nucEGFP gene body
489_2as	AGCTCGATGCGGTTCACCA	ChIP primer in nucEGFP gene body
738_1s	CACATGGTCCTGCTGGAGTT	ChIP primer in nucEGFP gene body and polyA
738_1as	ATCTAGAGTCGCGGCCGG	ChIP primer in nucEGFP gene body and polyA
IGR_5s	CGTGTCTGTCGGGGCTTTT	ChIP primer in intergenic region
IGR_5as	TGGGAGAGTAAAGTCAGAGAGG	ChIP primer in intergenic region
PVP298	CCCAACTTCTCAGGGACTGT	ChIP primer in promoter of CpGDep
PVP299	TCAATAGGGGTGACTAGTGGAGA	ChIP primer in promoter of CpGDep
PVP292	CCCACTGAACTCCCATTGAC	ChIP primer in promoter of CpGFull
PVP313	ATGCGGTTTTGGCAGTACA	ChIP primer in promoter of CpGFull

### Plasmid design and generation

The CpGDep and CpGFull expression plasmids were created by inserting the respective promoters into the BamHI site of a reporter with ZFHD1 and Gal4 DNA binding domains upstream of a nucEGFP (N261), using infusion cloning (Clontech). Balb/C-specific homology arms were amplified from genomic DNA of TC1 mouse ES cells. The csHP1α-tandem-FRB expression plasmid (P070) was generated by removing the puromycin resistance gene from a preexisting csHP1α-tandem-FRB expression plasmid (N163 [40], Addgene #44195) and replacing it with a hygromycin resistance gene. The Cas9 and sgRNA containing plasmid (P023) was created as described [43]. All cloning steps were performed in DH5α or One Shot Stbl3 cells (Invitrogen).

### Transformation of mouse embryonic fibroblasts (MEF)s

BALB/c 3T3 clone A31 cells (ATCC Number: CCL 163, passage 71) were grown in DMEM High Glucose with 10% Colorado Calf Serum and pen/strep at 37°C and 5% CO_2_. Cells were passaged every 2–5 days, and we kept between 30% and 80% confluency. Cells were transformed by the addition of lentiviral-delivered Large-T antigen (N234) and continually cultured until cells could be passaged at a 1:20 dilution (~3 weeks), which is a signature of transformation of MEF cells. Lines were then switched to fetal bovine serum-supplemented growth media, as previously described [44].

### Cell culture and CIP heterochromatization assay

Cells were grown in FBS-supplemented growth media (Gibco 26140–079, Lot #1972526) [44] and selected with 9 μg/ml blasticidin and 400 μg/ml hygromycin to drive csHP1α-FRB and FKBP-GAL4 expression. One day prior to experimentation, blasticidin and hygromycin were removed from the cells, which were then grown in drug free media. Rapamycin from a 10 μM stock dissolved in ethanol was added to media at 3 nM concentration.

#### Rapamycin washout and addition of 5-aza

Washout of rapamycin was performed by the addition of FK506 at 100 nM concentration for 48 hours, which competes with rapamycin at the active site of the FK506 binding protein (FKBP). Cells exposed to both FK506 and 5-aza simultaneously were exposed to 100 nM FK506 and 5 μM 5-aza for 48 hours, then FK506 was removed and the cells were exposed exclusively to 5-aza for an additional 72 hours.

### CRISPR/Cas9 insertion of expression constructs

Cell lines were created using the Cas9 double nuclease, essentially as previously described [43]. 2.5 μg of the Cas9 expression plasmid (P023) and 2.5 μg of one of the reporter plasmids (linearized with KpnI) were co-transfected into one million transformed MEFs using an Amaxa 4-D Nucleofector on program EH-100.

### Lentiviral infections

293T LentiX cells (Clontech) were transfected using polyethyleneimine (PEI), as described in [45]. Lentiviral infection of Large-T antigen did not require antibiotic selection, as transformed cells have a growth advantage and emerge from a sparsely populated plate where untransformed MEFs undergo senescence. Lentiviral infection of the clonal lines with the csHP1α-tandem-FRB and GAL4-FKBP fusion constructs required an outgrowth time of two days before selection with blasticidin (N118, Addgene #44245) or hygromycin (P070).

### Clonal isolation of cell lines

Transfected cells were seeded into a 96-well plate at a dilution of 80 cells per 10 mLs of growth media, with 100 μL distributed to each well. The cells were grown for two days, then each well was checked for individual cell plaques. Wells with single plaques were selected for expansion as clonal lines. Each line was checked for CRISPR insertion of the expression constructs using PCR and Southern blot analysis.

### PCR verification of inserts

Genomic DNA was isolated from each line using a previously described method [40]. Cells were digested with Proteinase K at 400 μg/ml at 56 °C overnight, then purified by phenol/chloroform extraction and ethanol precipitation. PCR verification of proper insertion was performed with a primer inside the nucEGFP of the expression construct and a primer outside of the 5’ homology arm.

### Southern blot determination of inserts

Genomic DNA was isolated from clonal lines and treated with Proteinase K at 400 μg/ml at 56 °C overnight, then purified by ethanol precipitation. Five μg of DNA for each clone was digested with EcoRI-HF for one hour and twenty minutes, before heat deactivation at 65 °C for 15 minutes. Samples were then loaded into a 0.6% agarose gel in TAE and run at 25 V for 10 hours. The probe was created using a PCR DIG Probe synthesis kit (Roche), and the blot was performed using a DIG Wash and Block Buffer set (Roche) and DIG Luminescent Detection Kit (Roche), as per the manufacturer’s instructions. The depurination step was not used. Carestream BIOMAX Light Film (Kodak 1788207) was exposed for 2 hours and developed with standard methods.

### FACS and flow cytometry

FACS of GFP-expressing cells for each cell line was performed by the UNC Flow Cytometry Core on a FACSARIAII (BD Biosciences). The flow cytometry was performed on two Attune Nxt machines (Thermo Fisher) in biological triplicate. Due to the differences in blue laser intensity, mean fluorescent intensity of GFP expression cannot be directly compared between Attune 1 and Attune 2. Samples were analyzed using FlowJo software. Sample gating can be found in S1 Fig.

### Chromatin immunoprecipitation (ChIP)

ChIP was performed using a modified version of a previously described protocol [46]. Briefly, 6 million cells were harvested as described above, and crosslinked with 1% formaldehyde for 10 minutes. Sonication was performed in 90 μl of Covaris Shearing buffer (0.1% SDS, 1 mM EDTA pH 8, 10 mM Tris HCl pH 8) and 10 μl of a nanodroplet cavitation reagent MegaShear (Triangle Biotechnology) as described [47] with a sonication time of 8 minutes at 200 cycles/burst to produce DNA fragments ~200-500bp. ChIP lysate equivalent to five million cells in Covaris Shearing buffer was adjusted with 5x IP Buffer (250 mM HEPES/KOH pH 7.5, 1.5 M NaCl, 5 mM EDTA, 5% Triton X100, 0.5% DOC, 0.5% SDS) to a salt concentration of 1x. 5 μg of H3K9me3 antibody (abcam ab8898) was added to adjusted lysate. 40 μL of Protein G Magnetic Dynabeads (Invitrogen cat. #10003D) were washed twice with 500 μL ChIP IP Buffer (50 mM HEPES/KOH pH 7.5, 300 mM NaCl, 1 mM DETA, 1% Triton X100, 0.1% DOC, 0.1% SDS), then resuspended in 40 μL ChIP IP Buffer. Dynabeads beads were added to the lysate/antibody mixture and incubated at 4°C overnight with end-over-end rotation. The next day, the beads were collected with a magnetic strip, then washed twice with 1 mL ChIP IP Buffer for 3 minutes each wash at room temperature with end-over-end rotation. The beads were then washed with 1 mL DOC buffer (10 mM Tris pH 8, 0.25 M LiCl, 0.5% TERGITOL, 0.5% DOC, 1 mM EDTA), then washed in 1mL TE pH 7.4. The beads were resuspended in 100 μl TE pH 7.4, supplemented with 2.5 μL SDS and 5 μL 10 mg/ml Proteinase K (Invitrogen 25530–031), then incubated without agitation overnight at 65°C. The next day, supernatant was collected from the beads, combined with one wash of 100 μL TE, and purified using the Qiagen MinElute PCR Purification Kit (Qiagen ref # 28006). Biological replicates were performed in duplicate.

### qPCR

qPCR reactions were performed using FastStart Universal SYBR Green Master Mix (Rox) (Roche 04913914001), 0.03–10 ng of template DNA, and 2.5 μM of each qPCR primer (Table 1). The reactions were performed in 384-well plates on a ViiA 7 qPCR machine (Applied Biosystems). The reaction parameters were the same as previously reported [45], and the CT values were normalized to an intergenic region (IGR) [44]. Biological replicates were performed in technical triplicate.

### Bisulfite sequencing

Bisulfite conversion of DNA was performed using the EpiTect Bisulfite kit (Qiagen), as per the manufacturer’s instructions. Up to two million cells were harvested per line for each condition. For the 5-week silenced samples, cells were harvested at the time of rapamycin removal. A portion of the bisulfite-converted DNA was amplified through PCR and cloned into plasmids using the Invitrogen topoisomerase cloning kit (Invitrogen K457502). The plasmids were transformed into One Shot Stbl3 cells, and single colonies were submitted for sequencing. Methylation patterns were analyzed using the online BISMA software [48].

## Statistical analysis

Statistical analyses were performed using GraphPad Prism 7 software. Replicates from flow cytometry and qPCR were subjected to unpaired t-tests, and statistical significance was determined using the Holm-Sidak method, with alpha = 0.05.

## Results

**Fig 1:**
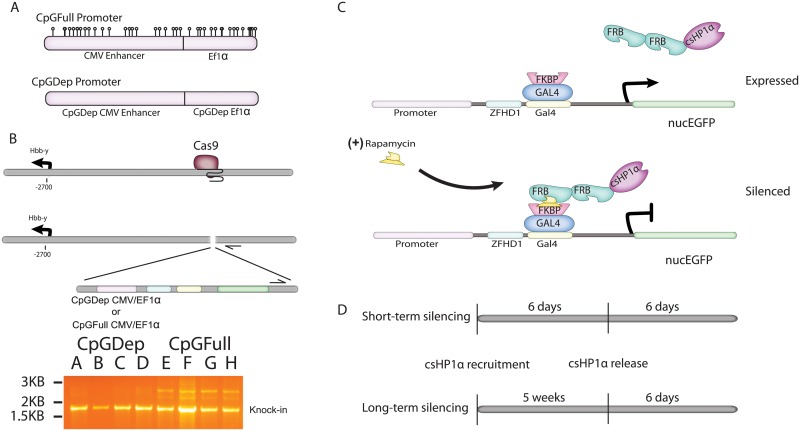
Creation of CpGDep and CpGFull reporter cell lines to test effects of CpG dinucleotide content on the kinetics of HP1-induced heterochromatization and gene silencing. A) The wildtype CMV/EF1α promoter has 39 total CpG dinucleotides, represented by lollipops, while the CpGDep is completely devoid of CpG sites. B) GFP-expression cassettes are driven by the CpGDep or CpGFull promoters inserted into an intergenic region outside of the Balb/C *β-Globin* locus using CRISPR/Cas9 targeted insertion. Verification of successful knock-in was performed by PCR. PCR primers are indicated by black arrows and can be found in Table 1. C) The CiA system uses CIP-rapamycin to recruit csHP1α to the reporter gene in a reversible manner. D) The timeline for “short-term” silencing is six days of csHP1α recruitment, followed by washout of rapamycin and addition of FK506. “Long-term” silencing is characterized by five continuous weeks of csHP1α recruitment before CIP washout.

In order to determine the role of CpG dinucleotides in the kinetics of gene silencing and heterochromatin maintenance, we designed two reporter gene constructs with promoters of different CpG content. The CpGFull promoter is comprised of a wild-type human CMV enhancer and core EF1α promoter sequence and contains 39 total CpG dinucleotides clustered closely together. The CpGDep promoter is comprised of a CpG-depleted mutant of a murine CMV enhancer and human EF1α promoter, of similar length and GC composition of the CpGFull promoter but devoid of CpG dinucleotides (Fig 1A and S2 Fig). We used CRISPR/Cas9 gene editing to insert a nucEGFP gene driven by the CpGFull or CpGDep promoter outside of the *Hbb-γ* gene in the *β-globin* locus in the mouse genome of large-T transformed MEFs [43]. We isolated four clonal cell lines for each construct and verified the successful genomic insertion with polymerase chain reaction (Fig 1B, Table 1). Additional insertions into the genome were identified through Southern Blot analysis (S3 Fig). We excluded the two clones that did show the predicted two kilobase band for our insertion and sorted the remaining six cell lines to have uniform GFP expression profiles (S4 Fig). Despite the additional genomic insertions, all cells in a population responded similarly to csHP1α recruitment followed by release. Therefore, these six lines are appropriate to use for the comparison of repression dynamics with these two different promoters. The three cell lines with the CpG-depleted promoter we called “CpGDep,” and the three cells lines with the wild-type promoter we called “CpGFull.”

We induced heterochromatinization of the reporter genes using the CIP-based Chromatin *in vivo* Assay (CiA) platform [40]. CiA utilizes CIP-rapamycin mediated recruitment of fusion proteins to reversibly tether an effector protein to a specific gene locus. The presence of Gal4 and ZFDH1 DNA binding sites in the reporter constructs allows recruitment of the chromoshadow of HP1α (csHP1α) to the nucEGFP gene in the presence of CIP-rapamycin (Fig 1C). CIP addition binds the FK506 binding protein (FKBP) fusion with GAL4 at the CiA locus and the FKBP–rapamycin binding (FRB) domain-tagged csHP1α. Following CIP addition, tethered exogenous csHP1α recruits endogenous HP1 proteins and HMTs to perpetuate a heterochromatin domain and silence expression of the reporter gene [40]. Because the recruitment is dependent on a small molecule bridge, it can be reversed by replacing rapamycin with FK506, which binds only to FKBP and not the FRB domain tag, helping to rapidly compete off rapamycin CIP and dislodging the initial csHP1α nucleation event. This reversibility allows us to investigate the durability of the induced heterochromatin domain after short-term and long-term silencing (Fig 1D). Once the nucleating csHP1α is removed, the heterochromatin domain is left subject to the natural cellular processes governing epigenetic memory.

**Fig 2:**
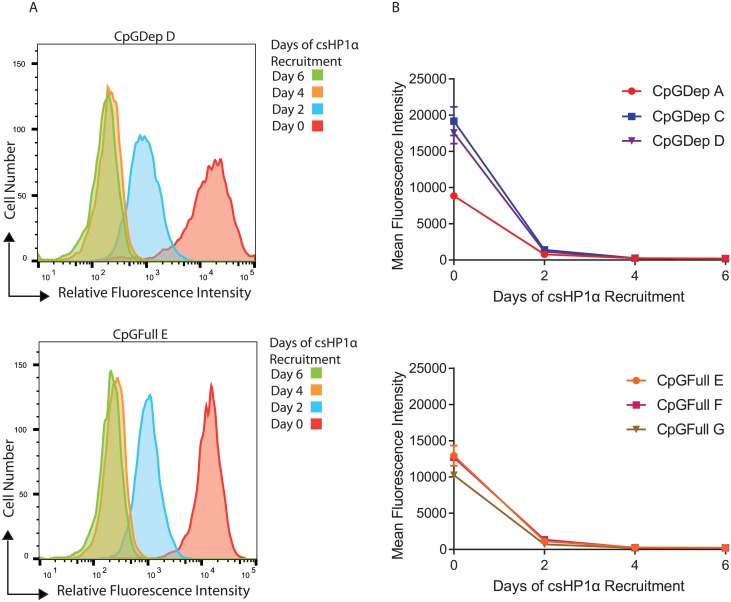
Recruitment of csHP1α induces silencing of GFP expression. A) Representative histograms show reduction of relative GFP levels as measured by flow cytometry in CpGDep D and CpGFull E cell lines during csHP1α recruitment. B) Mean fluorescent intensities of CpGDep and CpGFull clonal cell lines during csHP1α recruitment, averages of three biological replicates on Attune 1.

Induction of heterochromatin in the three clonal cell lines for each promoter was measured by the reduction of cellular green fluorescent protein (GFP) levels from the nucEGFP reporter gene. Flow cytometry analysis of a cell line over six days of csHP1α recruitment shows a ten-fold reduction of GFP after two days based on relative fluorescent intensity of individual cells in a population, and almost complete absence of GFP after six days (Fig 2A). We then averaged this intensity across all the cells in each sample to get the mean fluorescence intensity for that population. All six cell lines, CpGDep and CpGFull, followed this pattern of silencing over six days of csHP1α recruitment despite the fact that the CpGDep lines were expressing a slightly higher baseline of gene expression before silencing (Fig 2A & 2B). These data show that the two promoters types are repressed at similar time points after the CIP-rapamycin mediated recruitment of csHP1α, as measured by gross reduction of GFP levels by the second day.

**Fig 3:**
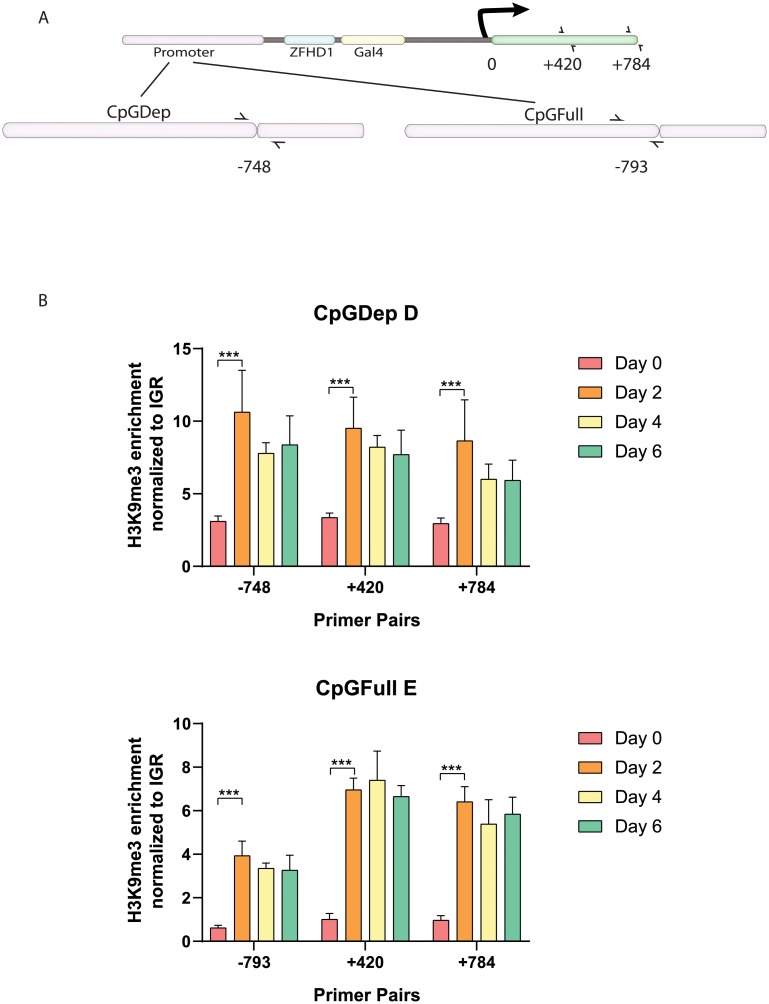
Enrichment of H3K9me3 in two cell lines during six days of CIP. A) Position of qPCR primers along gene body and promoters in CiA cell lines. Primers are depicted as black arrows, and primer sequences can be found in Table 1. B) Relative H3K9me3 enrichment for each primer set at days 0, 2, 4 and 6 post CIP. Each sample contains two biological and three technical replicates, n = 6. ** P ≤ 0.01. *** P ≤ 0.001. Significant difference between Day 0 and Day 4, and between Day 0 and Day 6, was also found in both cell lines, with all four primer sets. P≤0.001.

**Fig 4:**
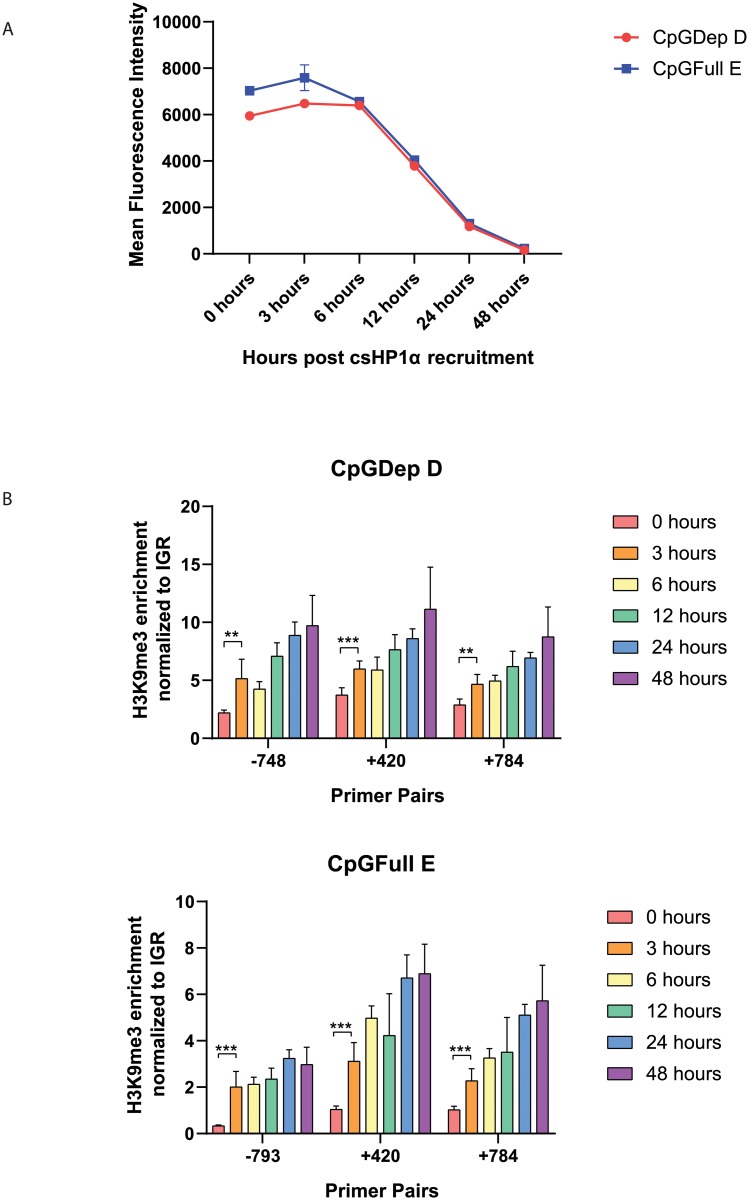
Gene silencing and enrichment of H3K9me3 in two cells lines during 48 hours of CIP. A) Reduction of GFP expression as measured by flow cytometry over a period of 48 hours in CpGDep D and CpGFull E cell lines on Attune 2. B) Relative H3K9me3 enrichment over the first 48 hours of CIP. qPCR primers are the same as used in Fig 3. Each sample contains two biological and three technical replicates, n = 6. ** P ≤ 0.01. *** P ≤ 0.001. Significant difference between 0 hours and all other timepoints was also found in both cell lines, with all four primer sets. P≤0.01.

In order to investigate if the different promoters contributed to different epigenetic architectures upon csHP1α-induced heterochromatization, we performed ChIP-qPCR for H3K9me3 enrichment after short-term silencing. We selected two regions along the nucEGFP gene body to compare, +420 and +784 bp downstream from the transcription start site (TSS) because these sequences were identical in both CpGDep and CpGFull lines. We also designed different primers for H3K9me3 enrichment in the promoters of each cell line (Fig 3A). We performed ChIP for H3K9me3 enrichment during the six days of csHP1α recruitment and found that for both CpGDep D and CpGFull E lines, H3K9me3 enrichment plateaus after 48 hours (Fig 3B). In order to capture the accumulation of H3K9me3, we performed an early time-course experiment, cataloging both gene silencing and H3K9me3 dynamics within the first 48 hours. In both the CpGDep D and CpGFull E lines, gene silencing is not measurably changed until 12 hours after CIP (Fig 4A). However, in both lines measurable H3K9me3 accumulation is seen as early as three hours post csHP1α recruitment, indicating a lag time between the recruitment of histone modifications and ability to measure the decrease of gene expression from the cassettes (Fig 4B).

**Fig 5:**
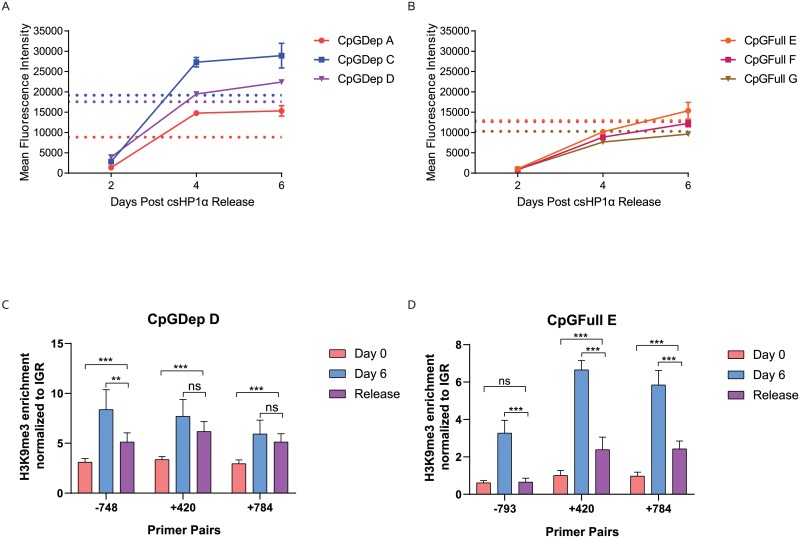
Recovery of gene expression after short-term heterochromatization. A) Re-expression of the silenced reporter in the CpGDep lines, as measured by relative GFP fluorescence on Attune 1 in biological triplicate, n = 3. Cells were harvested at days 2, 4 and 6 post csHP1α washout and analyzed by flow cytometry. Dotted lines represent baseline expression of GFP in each cell line not exposed to rapamycin. B) Same as A but using the CpGFull cell lines. C) Enrichment of H3K9me3 in CpGDep D cell line four days after CIP release compared to unsilenced and day 6 CIP. D) Enrichment of H3K9me3 in CpGFull E cell line four days after CIP release compared to unsilenced and day 6 CIP. qPCR primers are the same as used in Fig 3. Each sample contains two biological and three technical replicates, n = 6. ** P ≤ 0.01. *** P ≤ 0.001.

We next removed the initiating csHP1α tether event by washing out the rapamycin with the addition of 100 nM FK506 for 48 hours and performed flow cytometry to determine the speed of re-expression from each reporter construct. The CpGDep lines recovered from silencing rapidly, completely re-expressing GFP by day four post-rapamycin washout (Fig 5A). These lines responded to the release from heterochromatin by expressing levels of GFP greater than the baseline cell lines before silencing, as represented by the dotted horizontal lines. The CpGFull lines took a full six days to re-express GFP to the same levels as before heterochromatization (Fig 5B) and did not display the same overexpression behavior as the CpGDep lines. However, despite this small difference in re-expression velocity, both the CpGDep and CpGFull clones recovered from csHP1α-induced silencing within the span of six days, and neither expression cassette harbored any “heterochromatin memory” with prolonged gene repression from this short-term silencing. We performed ChIP four days following CIP-rapamycin washout and measured a reduction in overall H3K9me3, but not complete ablation of the mark after four days of release (Fig 5C & 5D). The CpGFull line showed a significant decrease in the enrichment of H3K9me3 for all three primer pairs tested (Fig 5D). For the CpGDep line, however, significant reduction of H3K9me3 enrichment was only measured in the promoter (Fig 5C). This decrease in H3K9me3 in the promoter is likely indicative of why the gene was re-expressed so quickly in both cell lines.

**Fig 6:**
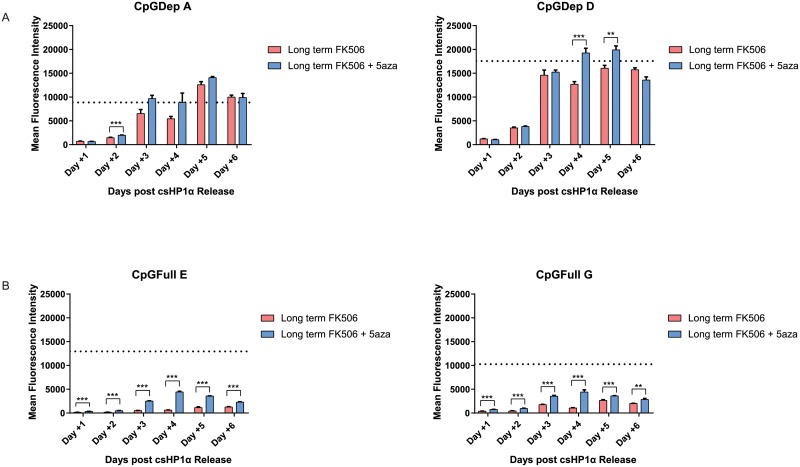
Recovery of gene expression after long-term heterochromatization. A) Re-expression of the CpGDep A and CpGDep D cell lines after five weeks of csHP1α-induced silencing, with or without the addition of 5-aza. B) Re-expression of the CpGFull E and CpGFull G cell lines after five weeks of csHP1α-induced silencing, with or without the addition of 5-aza. Dotted line represents baseline expression for each cell line. Flow cytometry was performed in biological triplicate on Attune 1. n = 3, ** P ≤ 0.01. *** P ≤ 0.001.

In order to allow for the engagement of the DNA methylation pathway and investigate the heterochromatin memory of the promoters, we recruited csHP1α in all six lines consecutively for five weeks [40]. We then performed a similar rapamycin washout as the short-term silencing experiment, but this time we compared the results of gene re-expression with and without the addition of the DNA methyltransferase inhibitor 5-azacytosine (5-aza) for the CpGDep and CpGFull lines. The CpGDep lines returned to almost baseline expression, with or without the addition of 5-aza, within six days of rapamycin washout (Fig 6A). The CpGFull lines, however, failed to return to baseline expression after six days of release (Fig 6B). The addition of 5-aza did improve the re-expression patterns in these cells but was not sufficient to re-establish baseline expression. In the two CpGFull lines tested the bulk mean fluorescent intensity was lower after stimulus release, suggesting that a durable gene repression was created in this system. Many cells in this HP1 release samples remained in a GFP-negative state for the duration of the experiment and remained 3.5–5.6-fold repressed on average after six days of csHP1α CIP-tether release.

We next performed bisulfite sequencing on a portion of the promoter regions of two CpGFull clones, which spanned 32 out of the 39 CpG dinucleotides in the CpGFull promoter (S5A Fig). After five weeks of CIP-HP1 induced silencing, CpGFull E displayed only 10% methylation of cytosines in this region, which remained stable even after CIP-HP1 washout (S5B Fig). However, the levels of DNA methylation were too low in these lines to make any real conclusions about the contribution of DNA methylation to the sustained silencing in these cells.

## Discussion

This work demonstrates the importance of the underlying genetic sequence and CpG dinucleotide content to the long-term stability of an HP1-mediated heterochromatin domain. The short-term silencing and heterochromatization of our reporter gene were unaffected by CpG dinucleotide content of the promoter, as evidenced by gene expression and H3K9me3 enrichment. Indeed, CpGDep and CpGFull reporter lines rebounded from heterochromatization within six days of csHP1α release, indicating no lasting effects of induced silencing. However, when continuously silenced for five weeks, the CpGDep line fully recovered expression levels to the same extent as before csHP1α recruitment, while CpGFull reporter lines were unable to recover and restart gene expression. As to why the CpGFull lines did not achieve full re-expression after the addition of 5-aza, it is possible that the experiment did not run long enough to see the return of GFP expression. If we had continued to monitor the cells after release, they may have eventually returned to baseline expression. However, levels of DNA methylation were too low for us to identify DNA methylation as causative in this case, and it is possible that DNA methylation is not the sole mechanism keeping these cells silenced. It will be interesting in the future to investigate what other epigenetic players might contribute to this heterochromatin memory.

Past experiments, mostly in the context of mESCs have shown an interdependence of histone and DNA methylation at regions of heterochromatin. For example, the DNA methylation at the pericentromere of mESCs is dependent upon the presence of the Suv39h histone methyltransferases, though the colocalization of HP1α/β with DNMT3a/b is not disrupted at the centromere of the Suv39h double null cells [8]. This may explain why bulk chromatin compaction is not lost in mESCs when DNA methylation is abrogated [49]. More prescient to our work, however, is the interaction between DNA methylation, or sequence, and histone methyltransferases in regions of traditional euchromatin, such as the direction of DNA methylation of the *Oct3/4* promoter by the HMT G9a, or the colocalization of SETDB1 and DNMT3a at the promoter of *p53BP2* in HeLa cells [50,51]. Because of the modular nature of this system, we can explore any region of the genome that may become silenced in a disease context.

Our work shows that csHP1α-induced heterochromatin cannot remain silenced at a controlled locus in the absence of CpG dinucleotides. In the past, many groups have used CpG-depleted transgenes in the hopes of evading DNA methylated-silencing by the cell [52,53], but those studies were focused on maintaining gene expression from the CpG-depleted genes, not on actively suppressing it to study HP1-specific heterochromatin memory as we can do by CIP-csHP1α recruitment. In our work, by intentionally inducing heterochromatin to two different promoter substrates, we can measure the specific contribution of DNA sequence to HP1-induced heterochromatin repression in the absence of other factors in a physiologically relevant setting. In this, we have created a powerful tool that can be used to further study the importance of underlying genetic sequence on the efficacy of other chromatin-modifying machinery.

Armed with this model system, we can explore the importance of time in establishing repressive domains. Because DNA methylation can become so misregulated in disease, there is a strong push to alter the chromatin landscape of endogenous genes with chromatin modifying machinery [54–57]. From our controlled system, we found that DNA sequence bears no impact on the speed with which you can initially induce a heterochromatin domain; csHP1α is well capable of establishing a repressive heterochromatin domain when CpG sites have been depleted from the promoter. However, to achieve durable repression after initial csHP1α stimulation, direct CpG methylation sites are required. We hope to use this new and modular system to explore other regulatory gene expression pathways in different genomic contexts in differentiated cells.

## Conclusion

In conclusion, we here provide evidence that the underlying genetic sequence itself can affect protein-driven epigenetic gene repression. We found that when CpG residues are removed from the immediate promoter region of a reporter transgene, CIP-mediated HP1 gene repression loses the ability to maintain durable gene silencing after release of the csHP1α initiating protein from the promoter by CIP washout. We also provide a new resource to study the contribution of DNA sequence to epigenetic transformations in the context of a living cell.

## Supporting information

S1 Fig Example gating of MEF cell lines in FlowJo software. Gating strategy, using CpGFull E cell line as an example on Attune 1. A) Forward scatter vs. side scatter to distinguish the live cell population. B) Forward scatter area vs. Forward scatter height to distinguish single cells from doublets. C) Blue laser channel area vs. violet laser channel area to exclude any cells that may be auto-fluorescing. D) A histogram of GFP expression measured in the blue laser channel.
(PDF)

S2 Fig Sequence alignments of the CpGFull and CpGDep promoters. A) Sequence alignments of the CMV enhancer portions of each promoter. B) Sequence alignments of the EF1α portions of each promoter. Identical base pairs are denoted by yellow.
(PDF)

S3 Fig Southern blot for genomic GFP insertions in eight clonal MEF cell lines. All eight original clonal lines were assayed for random insertion of the reporter constructs using a DNA probe against the gene body of nucEGFP. Genomic DNA was digested with EcoRI-HF, and probe detection was performed by DIG luminescence exposed to light film. Intended genomic insertion site is indicated at 2kb.
(PDF)

S4 Fig Pre-sort GFP expression of six clonal MEF cell lines. Pre-sort expression profiles of the six clonal lines chosen for this study. A narrow window of GFP expression was chosen (x-axis, GFP-A) in order to normalize GFP expression profiles for all six lines. The P4 for the CpGFull E clone also denotes GFP+ cells but was labeled differently because it was a separate sort session.
(PDF)

S5 Fig Bisulfite sequencing of the promoter region of two CpGFull clones. A) A region of 32 CpG dinucleotides denoted by red circles was analyzed for DNA methylation by bisulfite analysis. B) The DNA methylation profile of the CpGFull E clone repressed by CIP-csHP1α, after five weeks of silencing, after HP1 washout with FK506, and with the addition of 5-aza. C) CpGFull G. Red squares represent methylated cytosines. White square represents a mutated cytosine where DNA methylation state could not be determined. Percentages shown are percent methylation out of total potential sites.
(PDF)

S1 Table Balb/c homology arm sequence. Sequence of homology arms used in reporter constructs.
(PDF)
