# Supplementary figures and images for: Contribution of promoter DNA sequence to heterochromatin formation velocity and memory of gene repression in mouse embryo fibroblasts
Source: PLoS One. 2019 Jul 3;14(7):e0217699. doi: 10.1371/journal.pone.0217699 (PMC6608945; doi:10.1371/journal.pone.0217699)

A

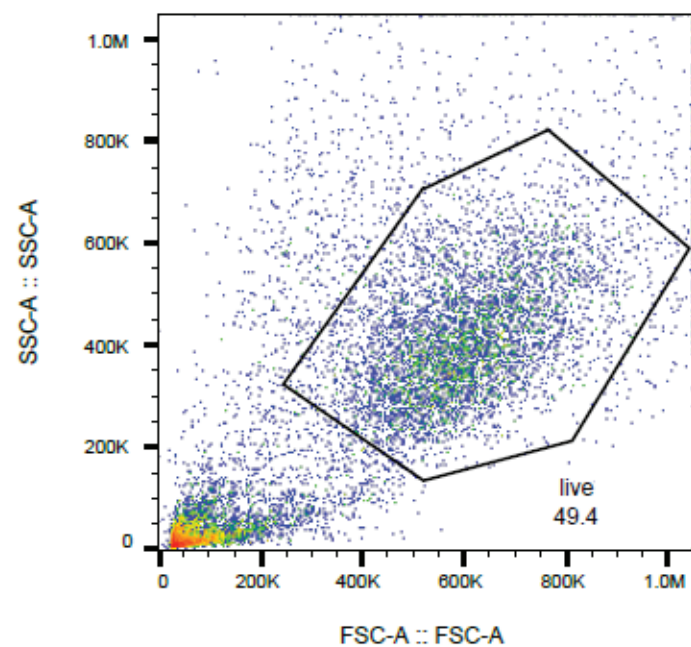

B

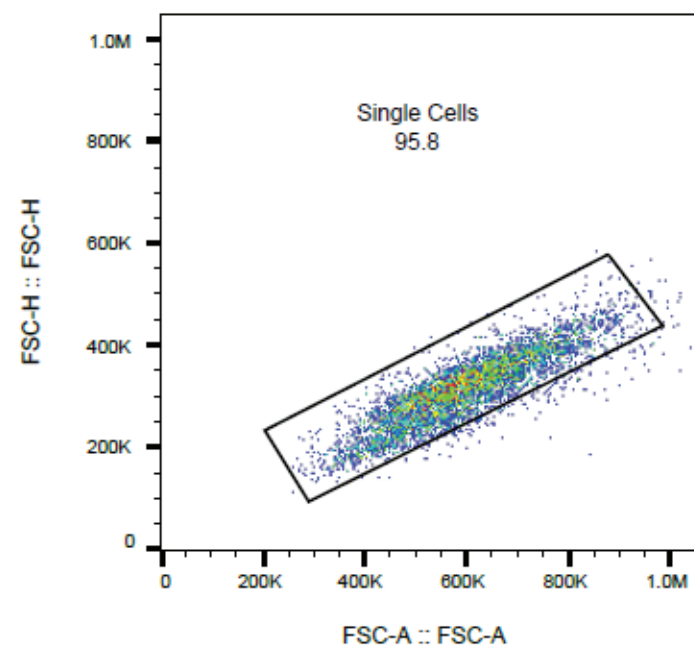

C

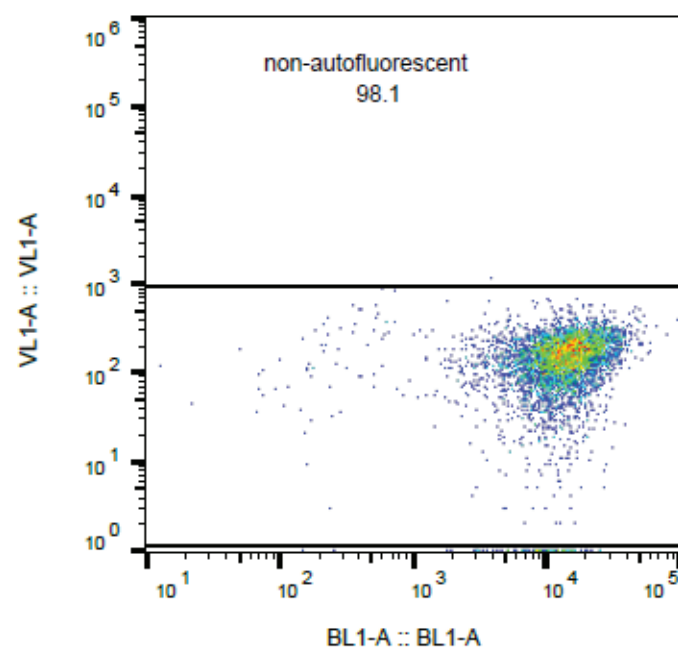

D

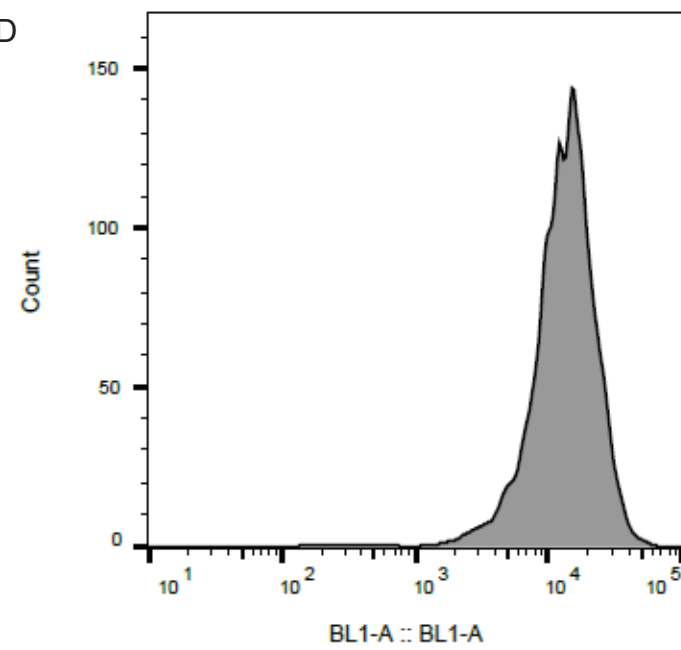

Supplement: S1 Fig — Gating strategy, using CpGFull E cell line as an example on Attune 1. A) Forward scatter vs. side scatter to distinguish the live cell population. B) Forward scatter area vs. Forward scatter height to distinguish single cells from doublets. C) Blue laser channel area vs. violet laser channel area to exclude any cells that may be auto-fluorescing. D) A histogram of GFP expression measured in the blue laser channel. (PDF) [file pone.0217699.s001.pdf]

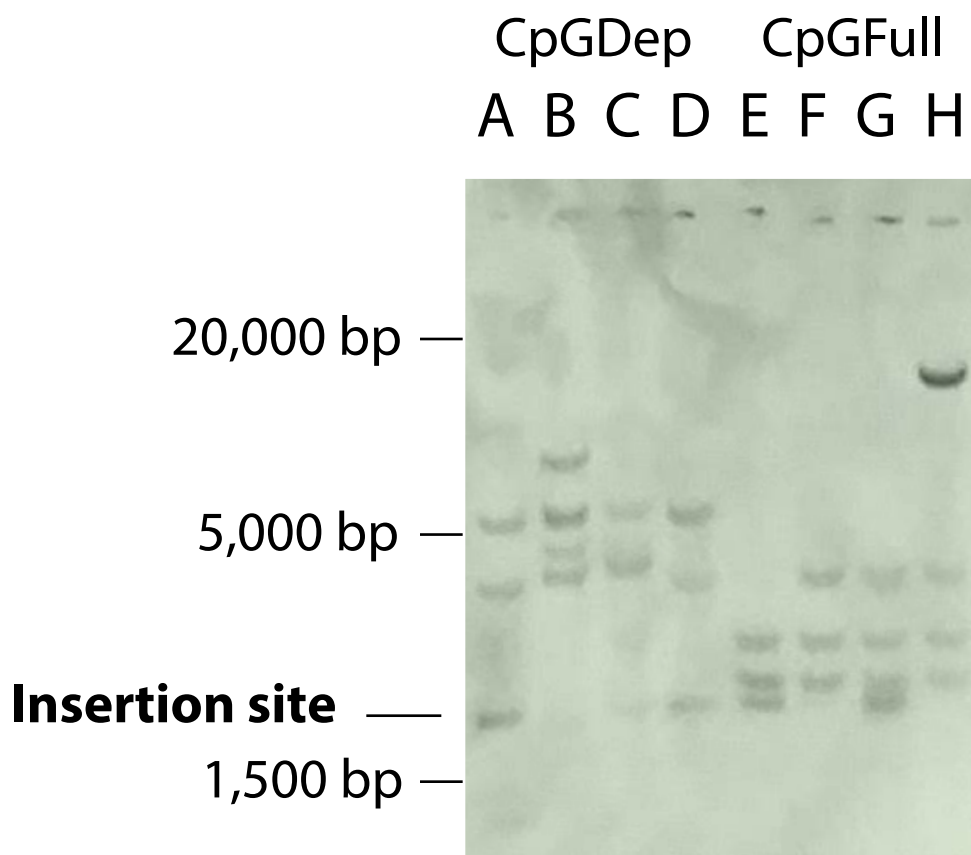

S3 Fig.

Supplement: S3 Fig — All eight original clonal lines were assayed for random insertion of the reporter constructs using a DNA probe against the gene body of nucEGFP. Genomic DNA was digested with EcoRI-HF, and probe detection was performed by DIG luminescence exposed to light film. Intended genomic insertion site is indicated at 2kb. (PDF) [file pone.0217699.s003.pdf]

CpGDep A

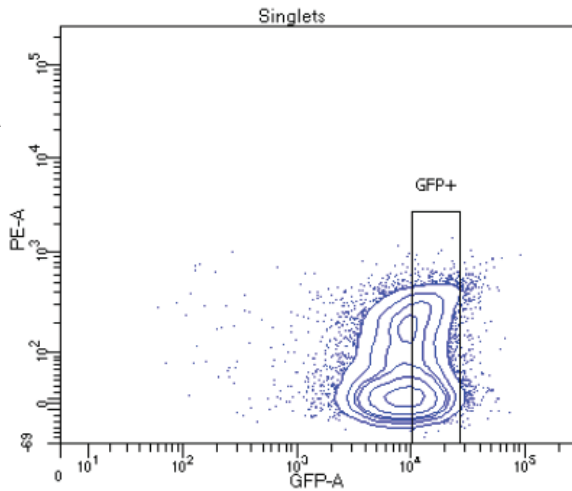

CpGFull E

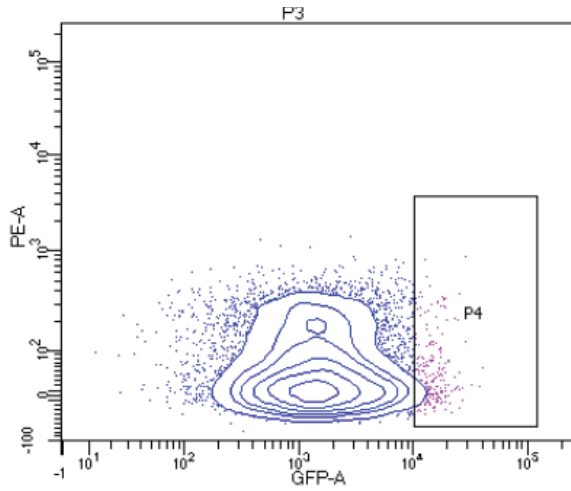

CpGDep C

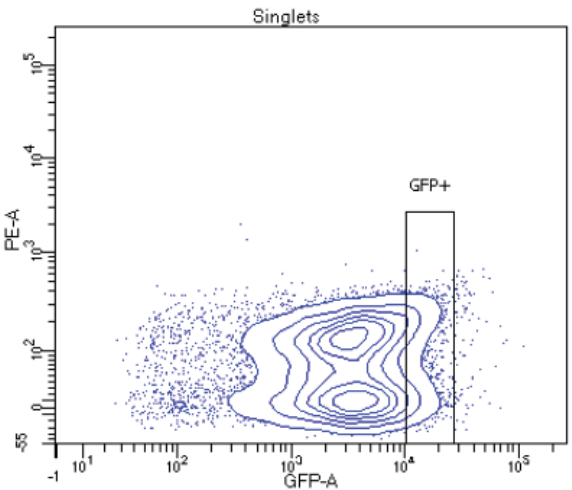

CpGFull F

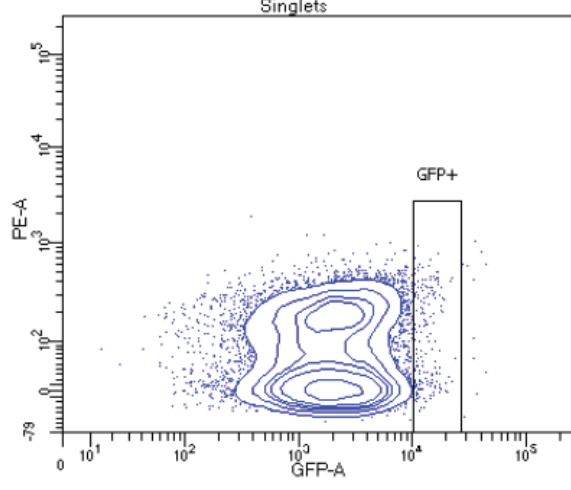

CpGDep D

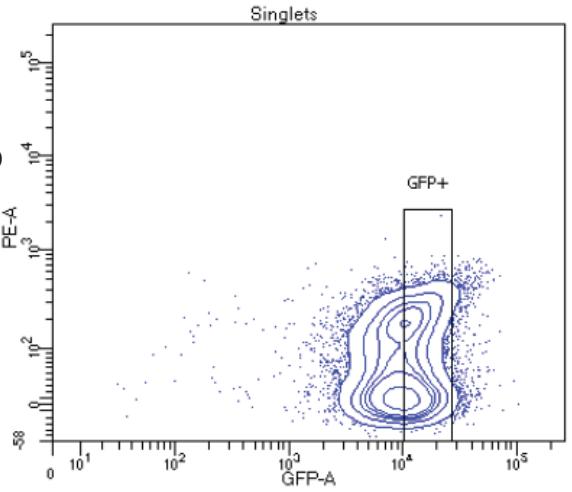

CpGFull G

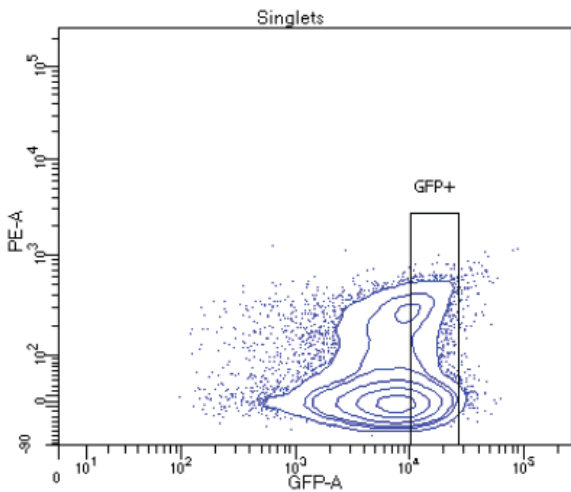

S4 Fig.

Supplement: S4 Fig — Pre-sort expression profiles of the six clonal lines chosen for this study. A narrow window of GFP expression was chosen (x-axis, GFP-A) in order to normalize GFP expression profiles for all six lines. The P4 for the CpGFull E clone also denotes GFP+ cells but was labeled differently because it was a separate sort session. (PDF) [file pone.0217699.s004.pdf]

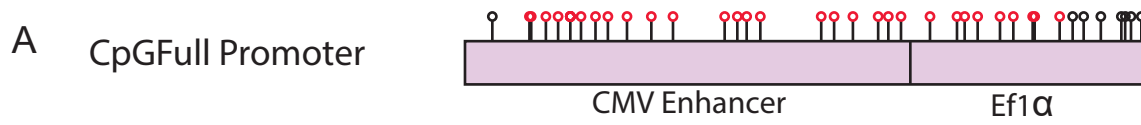

**B**

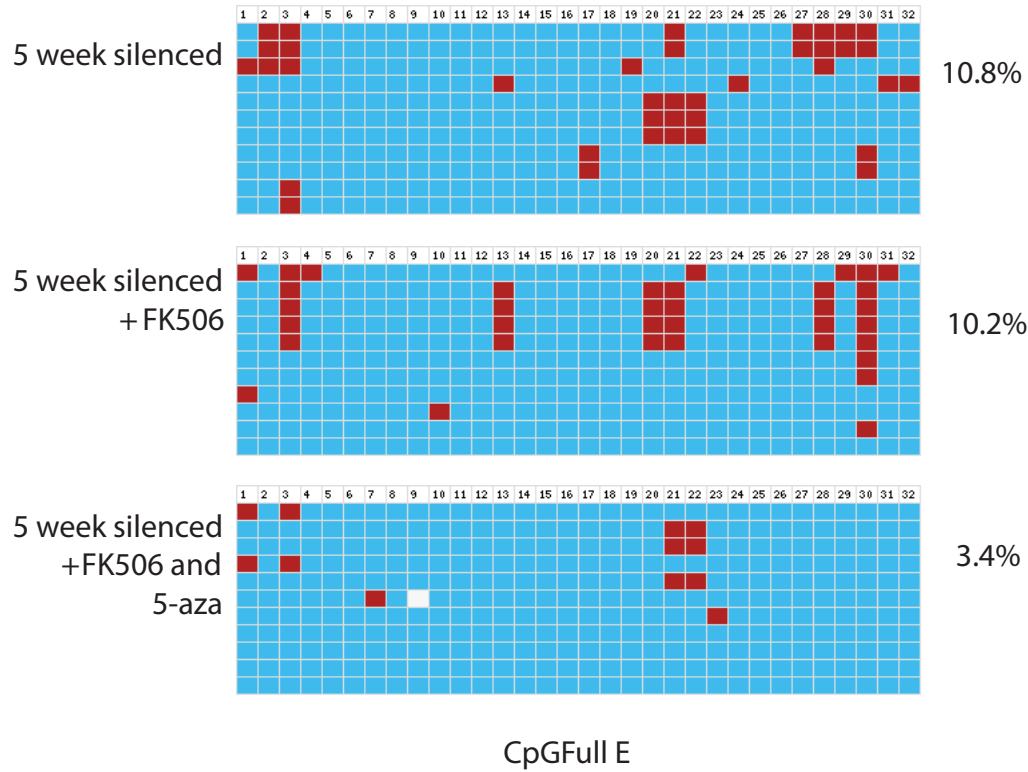

**C**

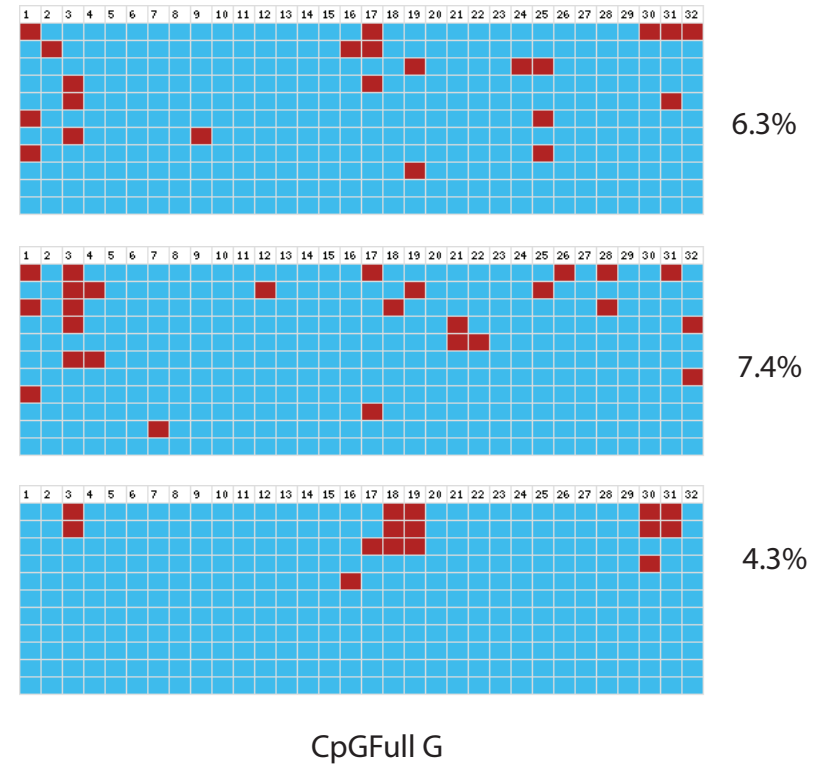

S5 Fig.

Supplement: S5 Fig — A) A region of 32 CpG dinucleotides denoted by red circles was analyzed for DNA methylation by bisulfite analysis. B) The DNA methylation profile of the CpGFull E clone repressed by CIP-csHP1α, after five weeks of silencing, after HP1 washout with FK506, and with the addition of 5-aza. C) CpGFull G. Red squares represent methylated cytosines. White square represents a mutated cytosine where DNA methylation state could not be determined. Percentages shown are percent methylation out of total potential sites. (PDF) [file pone.0217699.s005.pdf]
