# Supplementary material for: Contribution of promoter DNA sequence to heterochromatin formation velocity and memory of gene repression in mouse embryo fibroblasts
Source: PLoS One. 2019 Jul 3;14(7):e0217699. doi: 10.1371/journal.pone.0217699 (PMC6608945; doi:10.1371/journal.pone.0217699)
Supplement: S2 Fig — A) Sequence alignments of the CMV enhancer portions of each promoter. B) Sequence alignments of the EF1α portions of each promoter. Identical base pairs are denoted by yellow. (PDF) [file pone.0217699.s002.pdf]

A

```
                                                                         Section 1
                          (1) 1        10        20        30        40             55
CpGFull CMV Enhancer      (1) TAGTTATTAATAGTAATCAATTACGGGGTCATTAGTTCATAGCCCATATATGGAG
CpGDep CMV Enhancer       (1) GAGTCAATGGGAAAAACCCATT--GGAGCCA--AGTACACTGACTCAATAGGGAC
                                                                         Section 2
                         (56) 56            70        80        90        100      110
CpGFull CMV Enhancer     (56) TTCCGCGTTACATAACTTACGGTAAATGGCCCGCCTGGCTGACCGCCCAACGACC
CpGDep CMV Enhancer      (52) TTTC-CATTGGGTTTTGCCCAGTACATAAGGTCAATAGGGGGTGAGTCAACAGGA
                                                                         Section 3
                        (111) 111      120       130       140       150            165
CpGFull CMV Enhancer    (111) CCCGCCCATTGACGTCAATAATGACGTATGTTCCCATAGTAACGCCAATAGGGAC
CpGDep CMV Enhancer     (106) AAGTCCCATTGGAGCCAA-------GTACATTG----AGT-----CAATAGGGAC
                                                                         Section 4
                        (166) 166           180       190       200       210      220
CpGFull CMV Enhancer    (166) TTTCCATTGACGTCAATGGGTGGAGTATTTACGGTAAACTGCCCACTTGGCAGTA
CpGDep CMV Enhancer     (145) TTTCCAATGG-GT---TTTGCCCAGTACATAAGGTCAA---------TGGGAGGT
                                                                         Section 5
                        (221) 221      230       240       250       260            275
CpGFull CMV Enhancer    (221) CATCAAGTGTATCATATGCCAAGTAC-GCCCCCTATTGACGTCAATGACGGTAAA
CpGDep CMV Enhancer     (187) AAGCCAATGGGT--TTTTCCCATTACTGACATGTATACTGAGTCATTAGGG--AC
                                                                         Section 6
                        (276) 276           290       300       310       320      330
CpGFull CMV Enhancer    (275) TGGCCCGCCTGGCATTATGCCCAGTACATGA---CCTTATGGGACTTTCCT----
CpGDep CMV Enhancer     (238) TTTCCAA--TGGGTTT-TGCCCAGTACATAAGGTCAATAGGGGTGAATCAACAGG
                                                                         Section 7
                        (331) 331      340       350       360       370            385
CpGFull CMV Enhancer    (323) -ACTTGGCAGTACATCTACGTATTAGTCATCGCTATTACCATGGTGATGCGGTTT
CpGDep CMV Enhancer     (290) AAAGTCCCATTGGAGCCAAGTACACTGAGTCAATAGGGACTTTCCATTGGGTTTT
                                                                         Section 8
                        (386) 386           400       410       420       430      440
CpGFull CMV Enhancer    (377) TGGCAGTACATCAATGGGCGTGGATAGCGGTTTGACTCACGGGGATTT-CCAAGT
CpGDep CMV Enhancer     (345) GCCCAGTACA--AAAGGTCA---ATAGGGGGT-GAGTCAATGGGTTTTTCCCATT
                                                                         Section 9
                        (441) 441      450       460       470
CpGFull CMV Enhancer    (431) CTC--CACCCCATTGACGTCAATGGGAGTT
CpGDep CMV Enhancer     (394) ATTGGCACATACATAAGGTCAATAGGGGTG
```

B

```
                                                                            Section 1
                    (1) 1        10        20        30        40                58
CpGFull Ef1alpha    (1) ---------------------------------------CAGTGGCAGAGCGCACAT
CpGDep Ef1alpha     (1) ACTAGTGGAGAAGAGCATGCTTGAGGGCTGAGTGCCCCTCAGTGGCAGAGAGCACAT
                                                                            Section 2
                   (59) 59          70        80        90        100             116
CpGFull Ef1alpha   (20) CGCCCACAGTCCCCGAGAAGTTGGGGGGAGGGGTCGGCAATTGAACCGGTGCCTAGAG
CpGDep Ef1alpha    (59) GGCCCACAGTCCCTGAGAAGTTGGGGGGAGGGGTGGGCAATTGAACTGGTGCCTAGAG
                                                                            Section 3
                  (117) 117          130       140       150       160           174
CpGFull Ef1alpha   (78) AAGGTGGCGCG-GGGTAAACTGGGAAAGTGATGTCGTGTACTGGCTCCGCCTTTTTCC
CpGDep Ef1alpha   (117) AAGGTGGGGCTTGGGTAAACTGGGAAAGTGATGTGGTGTACTGGCTCCACCTTTTTCC
                                                                            Section 4
                  (175) 175  180       190       200       210       220       231
CpGFull Ef1alpha  (135) CGAGGGTGGGGGAGAACCGTATATAAGTGCAGTAGTCGCCGTGAACGTTCTTTTTCG
CpGDep Ef1alpha   (175) CCAGGGTGGGGGAGAACCATATATAAGTGCAGTAGTCTCTGTGAACATTC-------
```

S2 Fig.
