# Supplementary material for: Contribution of promoter DNA sequence to heterochromatin formation velocity and memory of gene repression in mouse embryo fibroblasts
Source: PLoS One. 2019 Jul 3;14(7):e0217699. doi: 10.1371/journal.pone.0217699 (PMC6608945; doi:10.1371/journal.pone.0217699)
Supplement: S1 Table — Sequence of homology arms used in reporter constructs. (PDF) [file pone.0217699.s006.pdf]

S1 Table

	Balb/c homology arm sequence
5’	gaccatggtgtccatgtcatacagactgaaagtaagcatatctgttacaactagagcataatattaaaaaaatgttctgtgggttcagtttaaagttttctcgcctagaatag<br>acagcctaggaaaatgtaactctagggtgagaaagatgttttgattgtttaagactgaatagacaaaaacagctggctgtcagcttgagtgcataacaagacccttgtttc<br>aaaaccccaagtctgagtgtttgcctagcatgtacaaagctctgggaatggattcaattcctatggctgctacaatctttttaaaagaaagaattcaatgctgtgctaatttt<br>agatgttattagtatagacatttttgaagtgtactgtttgtagtaaattgggcccttaaacacaagtttttcagtgttaaacaagaaaaaaaaatagtctctacagttgttctt<br>catcataaatagttttaaattagaaaaattgtgagtgaagtttctgaacaggaaaaacatgttttgcaattttattaaaagaaatggtcacttactctgcttccattgtatgtc<br>tccattcatattcccagaacagcaataaattagatagagtaagtctcttaggtgttgcattaactaggaatgactacatactaataacagcaaggtggcatccctgcagga<br>agccctaaaccttcaaggaacatgcaatggaaaatgtcaaagacactgaatgcctcagcctcatgatttgaagatgagttaaagagagagccatgtctttcttagctaga<br>aatccctcccttttcagt
3’	actgacctctggggctatactgaaaaaccagagaagtgtctggcacttaattatcacgagagtttttactaggaagagaaaatagaaaaccatagtcagccaggtattctc<br>ttgaaactggtaacctggaacaaagtcataagtgccctattttctcccataacatcacttctctttcttggtttctttggcatgttttaaaatgcttttaaaatgttaacattattg<br>ctacattgatatgtagaagtgtaaaacatttaaacaggttctccaaagcagcaaatatatatatatatattcccatctaaggcaggctacttgggtacttgatcaaaaggaa<br>acagaagatgagtcactggtctaagactacttcatttttgttaagctataatttgttgtcattatctaaattcaaacaaaacaagccagactgtttcttaaccctatttttacttt<br>ttcattggcagaaagctctcatacattctaggtaaaacaagtttctgcatttttacaatatcctataatagcttaccctatgatttatttatcatctccatggcattctttttaact<br>tgggtcctagagtaaataaattgtctattttcaggattaaaattctagagaactcttatctttttatgttaaataagctcacctgtgtattttataaatcaagatcaactcacca<br>aaacactctaacataataaaacacataggcctttttattaggtaggtactttatattttcaatattccccttcattttatactgcttcaatttataccttaaggagtagccaa
